# Combat Time in International Male Judo Competitions: A Systematic Review and Meta-Analysis

**DOI:** 10.3389/fpsyg.2022.817210

**Published:** 2022-03-08

**Authors:** Lindsei Brabec Mota Barreto, Marco A. Santos, Lucas O. Fernandes Da Costa, Diego Valenzuela, Felipe J. Martins, Maamer Slimani, Nicola L. Bragazzi, Bianca Miarka, Ciro José Brito

**Affiliations:** ^1^Department of Physical Education, Federal University of Juiz de Fora, Governador Valadares, Brazil; ^2^Department of Physical Education, Laboratory of Psychophysiology and Performance in Sports and Combats, Federal University of Rio de Janeiro, Rio de Janeiro, Brazil; ^3^Escuela de Kinesiologia, Universidad Santo Tomás, Santiago, Chile; ^4^Department of Physical Education, Federal University of Sergipe, Aracaju, Brazil; ^5^Department of Neuroscience, Rehabilitation, Ophthalmology, Genetics, Child and Maternal Health, Faculty of Medical and Pharmaceutical Sciences, University of Genoa, Genoa, Italy; ^6^Laboratory for Industrial and Applied Mathematics, Department of Mathematics and Statistics, University of Toronto, Toronto, ON, Canada

**Keywords:** time-motion studies, martial arts, athletic performance, psychomotor performance, task performance and analysis

## Abstract

This study aimed to synthesize literature data on male judo combat time in international competitions between 2010 and 2019. The search was carried out from May 8th to June 11th, 2021, in electronic databases using the following keywords: (“technical-tactical” OR “time motion” OR “combat time”) AND (“judo” OR “combat sports” OR “martial arts”). After the selection process, 8 articles were included in the systematic review and 7 in the meta-analysis. These studies analyzed 2,562 international male judo combats over the years 2010–2019. We observed that the average male judo combat time changed (2010 = 202.8; 2011–2012 = 304.8; 2016 = 237.4; 2018–2019 = 189.8 s) after each rule change (2010, 2013, 2017, and 2018). There was a significant difference between combats that ended up to the regular time and those that needed overtime (Golden Score: 2013 = 3% vs. 2018–2019 = 21%; *p* = 0.03). There were differences between 60 kg (*p* ≤ 0.019) and + 100 kg (*p* ≤ 0.04) categories and the others in 2011–2012. However, no significant difference was found between the combat time by weight division after the 2017 rule changes, although there are still differences in relation to the end of the combats (*p* < 0.001). There were significant changes in the male judo combat time with each rule change (2010, 2013, 2017, and 2018), and the data from the included studies point to a trend of homogeneity in the combat time spent between the weight divisions over the years, and an increase in the occurrence of Golden Score. More studies need to be carried out to identify the new temporal behaviors of athletes.

## Introduction

Since judo was introduced to the Olympics, its competition rules have gradually changed with each Olympic cycle (Franchini et al., 2013a; Dudeniene et al., 2017; Doppelhammer and Stockl, 2020). One of the main changes was the total male combat time (International Judo Federation, 2009, 2017a). The total combat time is composed of the regular combat time plus the overtime time (Golden Score, if the combat ends in a draw) and following the rules in force during the competition period. In addition to the current regular 5-min combat time for men, rule changes in 2010 reduced the Golden Score time from 5 to 3 min, and the *Koka* score was eliminated (International Judo Federation, 2009). The Golden Score time became unlimited from 2013 onwards; consequently, the *Hantei* (decision by a majority vote of the three referees) ceased to exist, and the punishments were no longer worth points to the opponent (International Judo Federation, 2013). The scientific paradigm for these alterations has been tested, and statistical evidence-based investigations could reveal a reverse predisposition of the rules changes, increasing the Golden scores (Stankovic et al., 2015, 2019; Calmet et al., 2017). Numerous judo coaches have experienced that best performance associated with psyching-up strategies on the physical and contextual training (Tod et al., 2015). Cognitive approaches are consistently related to behavior analysis and enhanced performance (results range from 61 to 65%) (Tod et al., 2015). Nevertheless, there is a lack of data for the practical application of sport psychology methods to acquire and retain new skills during judo combat time (Miarka et al., 2020b), as well as to modify behaviors that could reduce performance.

Systematic reviews and meta-analyses provide a method of pooling data from available primary combat time studies, exploring the moments, interventions, and weight division effects on a specific outcome in judo (Sterkowicz-Przybycien and Fukuda, 2014, 2016; Albuquerque et al., 2016). As such, systematic reviews and meta-analyses could support establishing sport psychology evidence-based guidelines and decision-making to effectively prescribe judo preparation or specific tactical training (Kashiwagura and Franchini, 2021). In this sense, identifying how combat time unfolds in the face of rule changes can help predict the time-motion analysis during combat.

Predominantly, time-motion analysis in judo goals to identify moments and frequency patterns (Castarlenas and Planas, 1997), often referred to as “performance indicators,” in the competitive environment (Ito et al., 2015; Klys et al., 2020; Barreto et al., 2021a). Though specific match demands have been well-described (Miarka et al., 2012; Challis et al., 2015; Dudeniene et al., 2017; Soriano et al., 2019; Brito et al., 2020), a practical guide of combat time analysis to verify how independent variables impact in judo is necessary to evaluate contextual evidence during judo tournaments (Tamura et al., 2012; Brito et al., 2017; Soto et al., 2020; Barreto et al., 2021). Previous research with match demands analysis on judo has discovered singular agents in the multifaceted strategic systems of high-level athletes (Calmet et al., 2006; Miarka et al., 2015; Del Vecchio et al., 2018), who have a predisposition to organize themselves into a vast array of synchronized patterns by altering their actions on the interactions with the opponent (Ito et al., 2014; Piras et al., 2014; Miarka et al., 2017). In addition, weight category differences in judo have been established, with heavyweights being predominantly susceptible to defeat after being penalized (Escobar-Molina et al., 2014; Sterkowicz-Przybycien et al., 2017) and lighter athletes producing more effective attacks when utilizing asymmetrical gripping strategies (Courel-Ibáñez et al., 2014; Kajmovic and Radjo, 2014; Kajmovic et al., 2014; Kons et al., 2018). Despite time-motion analysis having been habitually used within the study and applied situations to explore match demands (Brito et al., 2017a; Agostinho and Franchini, 2020), a meta-analysis of combat time associated with weight divisions and rule changes could increase the information that can potentially affect judo performance. This knowledge can provide statistical evidence for athletes’ physical and technical preparation (Marcon et al., 2010; Miarka et al., 2014; Julio et al., 2018; Samuel et al., 2019). Furthermore, this data could be employed in possible changes of evaluations, specific skills, and metabolic model demands (Miarka et al., 2012, 2018a; Ito et al., 2015, 2019; Franchini et al., 2018; Dal Bello et al., 2019).

Regarding rules change impact, the 2017 rules changes established that the regular combat time for men must reduce from 5 to 4 min, the *Yuko* score ceased to exist, the *Wazari* score no longer became *Ippon* score (accumulation of *Wazari*). The punishments no longer decided the winner of the regular combat (only on the Golden Score in case of more significant accumulation of punishments) (International Judo Federation, 2017a). In 2018, a new rule change determined that two *Wazari* scores would again become Ippon and that the punishment would no longer decide the winner in the Golden Score (International Judo Federation, 2017b). These constant rule changes were an attempt to promote the modernization of judo to make it more dynamic and televised (International Judo Federation, 2017a). Thus, the study of male judo combat time after these rule changes has been the focus of researchers. Ahmedov et al. (2020) carried out a study comparing combat time in different competitions, with differences between national and international levels. Their results showed that the aerobic capacity of athletes in international championships could be helpful to win matches. Soriano et al. (2019) analyzed the judo combat time phases and found that heterogeneity among athletes from different categories results in variability in combat times, which are directly affected by changes in the IJF rules. Therefore, they indicate that action and attack sequences could be used by trainers as a strategic plan and improve combat time management. Furthermore, knowing the temporal characteristics of combat and adapting the intensity and volume of technical training loads in judo, through the application of pedagogical means and methods, it is possible to obtain positive and specific results for the combat (Arziutov et al., 2016).

Thus, the present research carries out a systematic review and meta-analysis to understand how much combat time was spent in judo competitions between 2010 and 2019. We believe that the amount of time spent during combat, especially on the Golden Score, has changed over these years due to constant rule changes. Thus, this study aims to synthesize literature data on the male judo combat time in international competitions between 2010 and 2019 and weight division. This information will enable a better understanding of the effect of rule changes on the time needed to achieve victory in male judo combats, which will help coaches plan competition-specific training for each division.

## Materials and Methods

### Criteria for Considering Studies for This Review

Cross-sectional observational studies which analyzed the male judo combat time in international competitions were considered for this systematic review. These studies should contain data in seconds or minutes of the total male judo combat time in international competitions and identification of the year of the analyzed competition. For a secondary analysis, we observed whether the studies presented the total combat time separated by weight division [extra-lightweight (60 kg), half-lightweight (66 kg), lightweight (73 kg), half-middleweight (81 kg), middleweight (90 kg), half-heavyweight (100 kg), and heavyweight (+ 100 kg)].

The inclusion criteria for the studies were: (a) articles published in peer-reviewed journals; (b) studies written in Portuguese, English or Spanish. There was no limitation on the period for publication of articles because studies on this type of subject are recent. The exclusion criteria were: (a) studies that analyzed sports other than judo; (b) studies that performed an analysis in simulated combats; (c) studies whose analyzed combats are not from an international level; (d) studies whose sample consisted of adolescent athletes or just women; (e) articles that did not contain the total combat time.

### Search Methods for Identifying Studies

**FIGURE 1:**
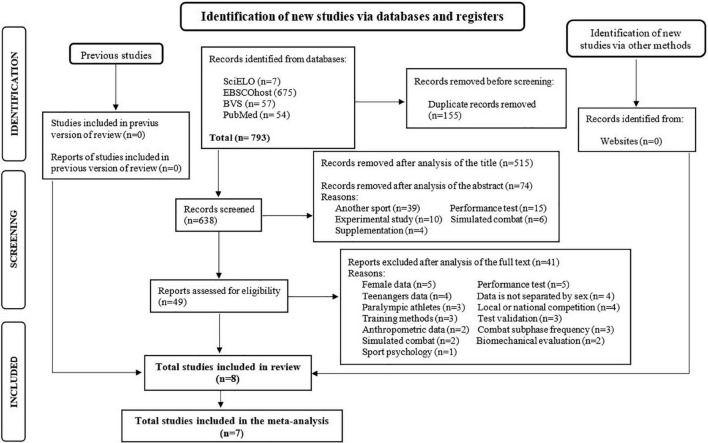
PRISMA flow diagram for study selection.

The search was carried out in the SciELO, PubMed, BVS—virtual health library (in LILACS, Medline, and IBECS bases), and EBSCOhost (in Sportdiscus, CINAHL, and Medline) electronic databases. The following keywords were used in the search process: (“technical-tactical” OR “time-motion” OR “combat time”) AND (“judo” OR “combat sports” OR “martial arts”). This search was carried out from May 8 to June 11, 2021. The references extracted from the databases were exported to the Excel 2013 program (Microsoft, Washington, United States). The entire article searches and selection process was conducted by two reviewers independently. Discrepancies between the two authors regarding the inclusion or exclusion of studies were resolved by consensus with a third author. The Preferred Reporting Items for Systematic Reviews and Meta-Analyses (PRISMA) (Liberati et al., 2009; Ardern et al., 2021) were followed in the screening process of articles collected in the databases. The article selection process is summarized in the PRISMA flow diagram (Figure 1).

A total of 793 articles were initially found in the search in the databases using the keywords. Of these, 155 duplicate articles were manually removed by the researchers. Next, 515 articles were excluded in the selection process by reading the title. These articles were removed because it was clear from the title that they analyzed athletes from other combat sports (Brazilian jiu-jitsu, taekwondo, boxing, mixed martial arts, etc.), or female judokas. Of the 123 remaining articles, 74 were excluded after reading the abstract. The reasons for exclusion were: analysis of other sports; analysis in simulated combat; experimental studies; analysis of performance tests or nutritional supplementation in judo. Thus, 49 articles were selected for analysis and assessment of eligibility for this systematic review. The authors analyzed the full text of the 49 articles and excluded 41 for the following reasons: studies with a sample consisting of Paralympic athletes, adolescents or only women; analysis of training methods, performance tests, anthropometric data, or biomechanical assessment; analysis of simulated combats, local or national competitions; studies for test validation; analysis of combat subphase frequency; studies on sports psychology; data analysis which was not separated by sex. Finally, 8 studies were included in this systematic review; however, only 7 studies were included in the meta-analysis because one of them did not have all the data needed for the analysis (Figure 1).

### Data Collection and Analysis

The quality of evidence was observed using GRADE (Grading of Recommendations, Assessment, Development, and Evaluations). GRADE has four levels of evidence—also known as certainty in evidence or quality of evidence: very low (i.e., The true effect is probably markedly different from the estimated effect), low (i.e., The true effect might be markedly different from the estimated effect), moderate (i.e., The authors believe that the true effect is probably close to the estimated effect), and high (i.e., The authors have a lot of confidence that the true effect is similar to the estimated effect) The following factors are considered to determine the level of evidence: (i) Study design; (ii) Methodological limitations (risk of bias); (iii) Inconsistency; (iv) Indirect evidence; (v) Inaccuracy; (vi) Publication bias; (vii) Effect magnitude; (viii) Dose-response gradient, and; (ix) Confounders. The present study used the free software GRADEpro to produce (Table 1).

**TABLE 1:** Certainty assessment, using GRADEpro.

Certainty assessment							Certainty
Study	Study design	risk of bias	Inconsistency	Indirect evidence	Inaccuracy	Other considerations	
Barreto et al. (2019)	Observational study	Not serious	Not serious	Not serious	Not serious	None	⊕⊕⊕⊕ High
Ceylan and Balci (2020)	Observational study	Not serious	Not serious	Not serious	Not serious	None	⊕⊕⊕⊕ High
Díaz-de-Durana et al. (2018)	Observational study	Not serious	Not serious	Not serious	Not serious	Strong association	⊕⊕⊕⊕ High
Segedi et al. (2014)	Observational study	Serious	Not serious	Not serious	Not serious	All potential confounders would reduce the demonstrated effect	⊕⊕⊕О Morate
Boguszewski (2016)	Observational study	Serious	serious	Not serious	Serious	All potential confounders would reduce the demonstrated effect	⊕⊕ОО Low
Adam et al. (2013)	Observational study	Not serious	Not serious	Not serious	Not serious	None	⊕⊕⊕⊕ High
Sterkowicz-Przybycien et al. (2017)	Observational study	Not serious	Not serious	Not serious	Not serious	Strong association	⊕⊕⊕⊕ High
Soriano et al. (2019)	Observational study	Not serious	Not serious	Not serious	Not serious	None	⊕⊕⊕⊕ High

*Relative and absolute effect sizes are available in the figures. As well as the risk of bias by the funnel plot. This table was produced using the GRADEpro software.*

After verifying the quality of evidence, the methodological quality of the eight studies included in the systematic review was assessed according to the RoBANS tool validated to analyze non-randomized studies. The Robans tool uses six risks of bias assessment items: selection of participants, confounding variables, exposure measurement, blinding of outcome assessment, incomplete outcome data, and selective outcoming reporting. These items should be classified as low, high, or unclear risk (Park et al., 2011). The risk of bias of the studies was independently performed by two reviewers; discrepancies were resolved by consensus with a third reviewer. The extraction of qualitative and quantitative data from the included studies was independently performed by at least two authors. The extracted data were entered into the Excel 2013 program (Microsoft, Washington, United States). Then, the following information was extracted for qualitative analysis of articles and presented in a table: (a) author and year of publication; (b) year of competition of the sample combats; (c) type of competition evaluated; (d) level of athletes; (e) combat time sample; (f) combat analysis instruments; (g) analysis protocol; (h) data extracted for this review. Next, the following information was extracted for the quantitative analysis of the included articles and presented in figures: (a) mean and standard deviation of total combat time in seconds; (b) frequency of combats that ended in regular time or in Golden Score; (c) total combat time by weight division.

Descriptive tables, graphs and forest plots were used to show the data analysis. The 2020 software Revman.5.4.1 (Review Manager, Version 5.4.1, The Cochrane Collaboration, London, United Kingdom) was used for the meta-analysis, considering a significance level of *p* < 0.05. A meta-analysis of dichotomous outcomes was used to verify the effect size of combats which ended in regular time vs. Golden Score time. This analysis used a random effects model and employed the Mantel-Haenszel statistical method with the odds ratio effect measure. A meta-analysis of continuous outcomes was used to verify the effect size between weight division based on the inverse variance statistical method with a random effects analysis model and standard mean difference of effect measure. The heterogeneity among included studies was assessed using Cochran’s *Q*-test and I^2^ statistic, and it was classified in: might not be important (0–29%), may represent moderate (30–49%), substantial (50–74%), or considerable (75–100%) heterogeneity (Higgins et al., 2021).

## Results

### Description of Studies and Quantitive Data

The 8 studies that met the eligibility criteria in this systematic review were: Adam et al. (2013), Segedi et al. (2014), Boguszewski (2016), Sterkowicz-Przybycien et al. (2017), Díaz-de-Durana et al. (2018), Barreto et al. (2019), Soriano et al. (2019), and Ceylan and Balci (2020). Table 1 shows the qualitative analysis of these articles. Only data referring to the duration of male combats were extracted from the studies in this systematic review, although some studies present female data and other variables, such as characteristics of combat phases, techniques used and attack effectiveness index. Therefore, the information described in Table 1 only refers to the data analyzed in this review.

The judo combats analyzed by the studies covered the period from 2010 to 2019, and involved the Olympic Games (2012; 2016), the World Championship (2018; 2019), and other international competitions involved in the World circuit such as the Grand Slam, Grand Prix, and others (2010–2013). A total of 2,562 male combats were analyzed over the years (2010 = 75; 2011–2012 = 1,356; 2013 = 125; 2016 = 7; 2018–2019 = 999). Two studies performed different analyzes of the same combats [Barreto et al. (2019): total combat time and its phases; and Díaz-de-Durana et al. (2018): total combat time and its phases by weight division]; therefore, we counted the amount of 548 combats only once in the sum of the total in this review (Table 2).

**TABLE 2:** Studies on time-motion analysis in male judokas in international competitions (*n* = 8).

Author	Competition year	Championships	Study group for this review	Combat time sample	Instruments	Protocol	Data for this review
Adam et al. (2013)	2012	London olympic games	Gold medal at the Olympic Games	35 combats 5 by each weight division	Standardized audiovisual techniques and graphic markings	Time motion indicators	Combat time**** Combat time by weight division
Barreto et al. (2019)	2011–2012	International (different competitions)	Athletes ranked for the olympic games	548 combats 60 kg *n* = 44; 66 kg *n* = 132; 73 kg *n* = 71; 81 kg *n* = 152; 90 kg *n* = 42; 100 kg *n* = 35; >100 kg *n* = 72	VirtualDub Program 1.8.6 Frami software	Combat phases	Combat time
Boguszewski (2016)	2016	Rio de Janeiro olympics games (Finals)	Gold and silver medal at the Olympic Games	7 combats 1 by each weight division	Observational sheets in 10-second fight sequences	Kalina’s method of combat dynamics measurement, with author’s modification	Combat time
Ceylan and Balci (2020)	2018–2019	World Championships	International level athletes	999 combats 60 kg *n* = 147; 66 kg *n* = 173; 73 kg *n* = 173; 81 kg *n* = 150; 90 kg *n* = 150; 100 kg *n* = 119; >100 kg *n* = 87	Data from official IJF website	Combat time	Combat time**** End of combat time Combat time by weight division
Díaz-de-Durana et al. (2018)	2011–2012	International (different competitions)	Athletes ranked for Olympic Games	548 combats 60 kg *n* = 44; 66 kg *n* = 132; 73 kg *n* = 71; 81 kg *n* = 152; 90 kg *n* = 42; 100 kg *n* = 35; >100 kg *n* = 72	Frami software	Combat phases	Combat time by weight division
Segedi et al. (2014)	2013	Rijeka Grand Prix (elimination rounds)	International level athletes	125 combats 60 kg *n* = 15; 66 kg *n* = 23; 73 kg *n* = 17; 81 kg *n* = 24; 90 kg *n* = 17; 100 kg *n* = 14; >100 kg *n* = 15	Recorded by video camera	Combat end time and score analysis	End of combat time End of combat time by weight division
Soriano et al. (2019)	2010	International (different competitions)	International level athletes	75 combats 60 kg *n* = 25; 66+73+81 kg *n* = 25; >100 kg *n* = 25	Observation tool combined with a field format category system. LINCE v. 1.1	Time motion indicators	Combat time
Sterkowicz-Przybycien et al. (2017)	2011–2012	International (different competitions)	Athletes ranked for Olympic Games	773 combats 60 kg *n* = 77; 66+73+81 kg *n* = 412; 90+100 kg *n* = 155; >100 kg *n* = 129	VirtualDub Program 1.8.6 Frami software	Combat phases	Combat time by weight division

*kg, kilograms.*

The athletes analyzed in the combats studied were Olympic Games gold or silver medalists (Adam et al., 2013; Boguszewski, 2016) or they were classified for the Olympic Games (Sterkowicz-Przybycien et al., 2017; Díaz-de-Durana et al., 2018; Barreto et al., 2019), or they were international level athletes (Segedi et al., 2014; Soriano et al., 2019; Ceylan and Balci, 2020). This guarantees the high-performance level of the combats analyzed. The instruments and protocols used to assess the combats varied: observation instruments were used in spreadsheets analyzing 10-s fight sequences; standardized audiovisual techniques with graphic markings; an observation tool combined with field format category system; and the Frami, LINCE v. 1.1 and VirtualDub Program 1.8.6 software programs (Table 2). However, Calmet et al. (2019) found identical and stable analyses when expert judo performance analysts performed video analyses in slow motion.

### Risk of Bias and Quality of Evidence in Included Studies

**FIGURE 2:**
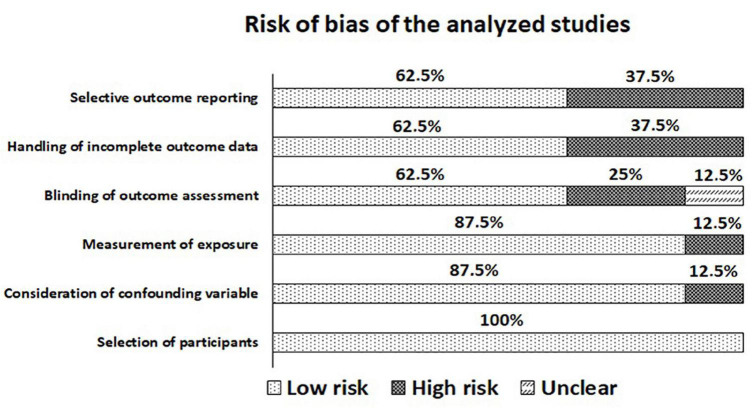
Review of author’ judgments about each risk of bias item across all included studies (%).

Figure 2 shows the authors’ judgments regarding the risk of bias of the 8 studies included in this systematic review, while the figure shows each RoBANS tool item. At least 62% of the articles included had a low risk of bias. However, ∼37% of the studies reported selective outcomes and handled incomplete outcome data. This happens because a large number of variables are collected when analyzing judo combats and led to several studies, and it would be impossible analyze all the variables in a single article in depth. About 25% of the articles were evaluated with problems in the blindness of outcome assessment, and this item was not clear in 12%. Some articles did not specify characteristics of the combat evaluators (judo graduation, competitive experience, and others), nor reliability tests for their analysis and/or instruments used. Finally, ∼12% of the articles had failures in measuring exposure and considering confounding variables. In these cases, the number of combats per category was not reported in the analysis separated by weight division and the authors did not discuss all of the data.

**FIGURE 3:**
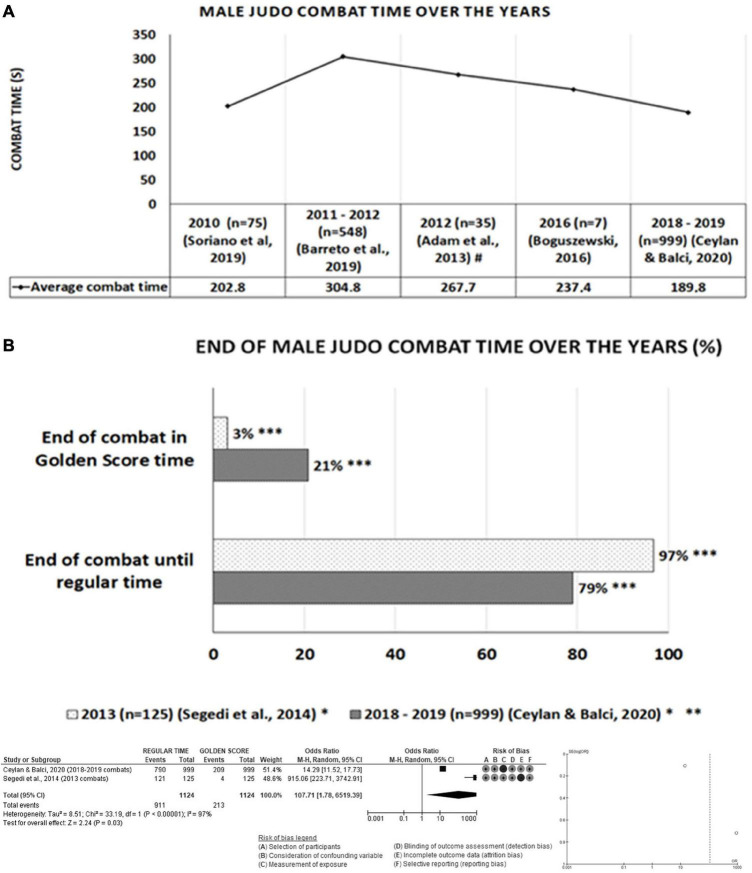
Total time and moment when male judo combats ended between 2010 and 2019. ^#^Average combat time calculated by the authors of this study based on the time data in the article; *percentage of combats calculated by the authors of this study; **we added up the combats which ended in 61–120, 121–80, 181–239, and 240s; ***significant difference (*Z* = 2.24; *p* = 0.03).

Figure 3 displays the quality of evidence, using GRADE (Grading of Recommendations, Assessment, Development, and Evaluations), which is a transparent framework for presenting summaries of evidence and provides a systematic approach for making practical recommendations.

### Quantitative Data on Male Combat Time

Table 3 shows the combat time data of the included studies. The average combat time from the study by Adam et al. (2013) was calculated by the authors of this study (based on existing data in the article). The average of the total combat time of male judo ranged between 304.8 ± 169.6 s (2011–2012) and 189.8 ± 105.9 s (2018–2019). The lowest average value by weight division was in 2010 in the 60 kg class (173.8 ± 91.1 s), and the longest combat time was in 2011–2012 in the 73 kg class (344.4 ± 191.6 s).

**TABLE 3:** Total combat time in male judo competitions between 2010 and 2019.

Weight division	Combat time (s) (mean ± standard deviation)						Combat time (s) (median; interquartile range)
		
	Soriano et al. (2019)	Barreto et al. (2019)	Díaz-de-Durana et al. (2018)	Adam et al. (2013) *	Boguszewski (2016)	Ceylan and Balci (2020)	Sterkowicz-Przybycien et al. (2017)
	
	*2010 International competitions (n = 75)*	*2011–2012 International competitions (n = 548)*	*2011–2012 International competitions (n = 548)*	*2012 Olympic Games (n = 35)*	*2016 Olympic Games (n = 7)*	*2018–2019 World Championships (n = 999)*	*2011–2012 International competitions (n = 773)*
All categories	202.8 ± 86.2	304.8 ± 169.6	–	267.7 ± 112	237.4	189.8 ± 105.9	–
60 kg	173.8 ± 91.1	–	198.6 ± 147.4	218 ± 216.9	–	187.3 ± 101.4	227; 213.8
66 kg	233.3 ± 78.2		300.1 ± 166.3	260.4 ± 133		190.9 ± 112	288.6; 205.7
73 kg			344.4 ± 191.6	265.2 ± 105.3		189.3 ± 112.7	
81 kg			330.6 ± 173.4	300 ± 0		187.1 ± 94.6	
90 kg	–		276.9 ± 158.9	280.2 ± 49.5		192.1 ± 111.2	327.3; 178.5
100 kg			323.9 ± 145.3	258.2 ± 150.5		194.1 ± 103.5	
+100 kg	201.5 ± 81.3		303.9 ± 155.5	291.8 ± 12.7		188.1 ± 100.5	219.6; 234.3

*Average combat time calculated by the authors of this study based on the time data in the article. s, seconds.

The moment when the combats end (before or in regular time, or Golden Score) is described in Table 3. In the study by Ceylan and Balci (2020), the data from the combats which ended in 61–120 s; 121–80 s and 181–239 s were added up to determine the value of the variable “Before regular time.” The percentage data of the end of combat time in Table 4 were also calculated by the authors. Most of the combats analyzed in both 2013 and 2018–2019 ended before the regular combat time (2013 = 63.2%; 2018–2019 = 64.7%). In the analysis by weight division in 2013, the 81 kg category had the most combats finished before regular time (79.2%); the 66 kg category had more combats finished at regular time (47.8%); and the 73 kg category had more combats that needed a Golden Score to define the winner (11.8%). Ceylan and Balci (2020) reported a significant difference in the relationship between weight division and end-of-combat time in 2018–2019 (χ^2^ = 2031.57; *p* < 0.001; PHI = 0.104). Nevertheless, different from 2013, the frequency of the Golden Score was higher in the 66 and 81 kg categories in 2018–2019 and continued to show the lowest occurrence of the Golden Score; however, the authors did not show these values by division.

**TABLE 4:** Moment when combat ends in male judo competitions over the years.

Weight division	End of combat time	Segedi et al. (2014)		Ceylan and Balci (2020)	
		*2013 Grand Prix (n = 125)*		*2018–2019 World Championships (n = 999)*	
		u	% #	U	%
All categories	*Before regular time*	79	63.2	646*	64.7^#^
	*In regular time*	42	33.6	144	14.4
	*Golden Score*	4	3.2	209	20.9
60 kg	*Before regular time*	10	66.7		
	*In regular time*	5	33.3		
	*Golden Score*	0	0		
66 kg	*Before regular time*	11	47.8		
	*In regular time*	11	47.8		
	*Golden Score*	1	4.4		
73 kg	*Before regular time*	7	41.2		
	*In regular time*	8	47		
	*Golden Score*	2	11.8		
81 kg	*Before regular time*	19	79.2		
	*In regular time*	4	16.6		
	*Golden Score*	1	4.2		
90 kg	*Before regular time*	10	58.8		
	*In regular time*	7	41.2		
	*Golden Score*	0	0		
100 kg	*Before regular time*	10	71.4		
	*In regular time*	4	28.6		
	*Golden Score*	0	0		
+100 kg	*Before regular time*	12	80		
	*In regular time*	3	20		
	*Golden Score*	0	0		

*Sum of the data present in the study for combats that ended in 61–120; 121–80; and 181–239 s; ^#^Percentage of combats calculated by the authors of this study. u, unit.

In view of the collected data, the studies were grouped for quantitative analysis as follows: (a) total combat time (Segedi et al., 2014; Boguszewski, 2016; Barreto et al., 2019; Soriano et al., 2019; Ceylan and Balci, 2020), whose distribution of combats was: 2010 = 75, 2011–2012 = 583, 2013 = 125, 2016 = 7, 2018–2019 = 999 (total = 1,789 combats); (b) total combat time by weight division (Adam et al., 2013; Díaz-de-Durana et al., 2018; Ceylan and Balci, 2020), whose distribution of combats was: 60 kg = 196, 66 kg = 310, 73 kg = 249, 81 kg = 307, 90 kg = 197, 100 kg = 159, + 100 kg = 164 (total = 1,582 combats) (Table 1). The study by Sterkowicz-Przybycien et al. (2017) presented their data as median and interquartile range (and not as mean and standard deviation) (Table 2), so we could not group it with the other studies for the quantitative analysis. Thus, only 7 studies were included in the meta-analysis.

#### Total Male Judo Combat Time Between 2010 and 2019

Figure 3A shows the total time in seconds of judo combats between 2010 and 2019, and Figure 3B the analysis of the moment when the combats ended in 2013 vs. 2018–2019. To do so, we summed the combats which ended in 61–120, 121–80, 181–239, and 240 s from the study by Ceylan and Balci (2020), and we calculated the percentage of data from the studies by Segedi et al. (2014) and Ceylan and Balci (2020). The meta-analysis showed a significant difference in the occurrence of Golden Score between the years 2013 vs. 2018–2019 (*Z* = 2.24; *p* = 0.03), as there was a 17.7% increase in the number of combats that needed overtime to define the winner in 2018–2019.

#### Total Male Combat Time by Weight Division Between 2010 and 2019

**FIGURE 4:**
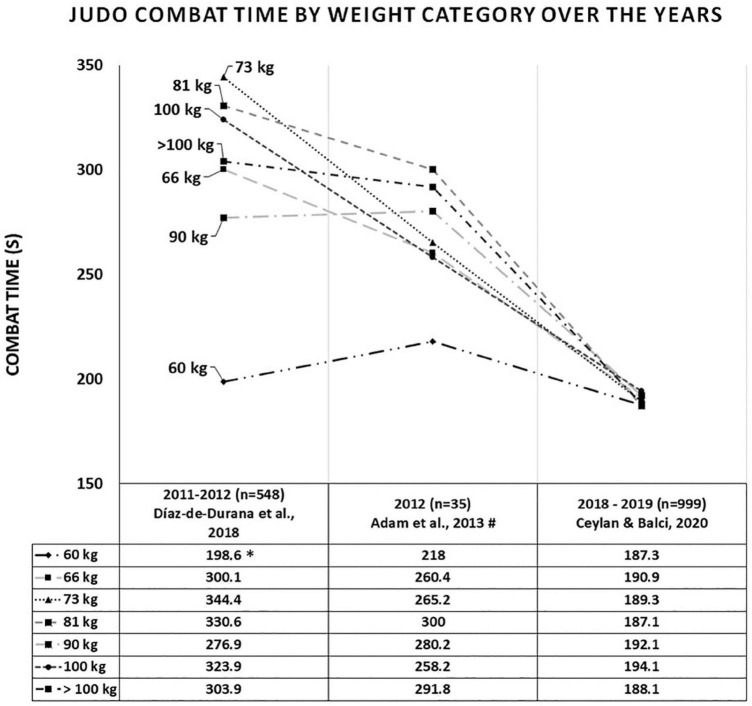
Duration of male judo combat by weight division over the years. *Significant difference (*p* = 0.001) between the 60 kg and the other categories (except the 90 kg category) in the study by Díaz-de-Durana et al. (2018): ^#^Average combat time by category calculated by the authors of this study.

**FIGURE 5:**
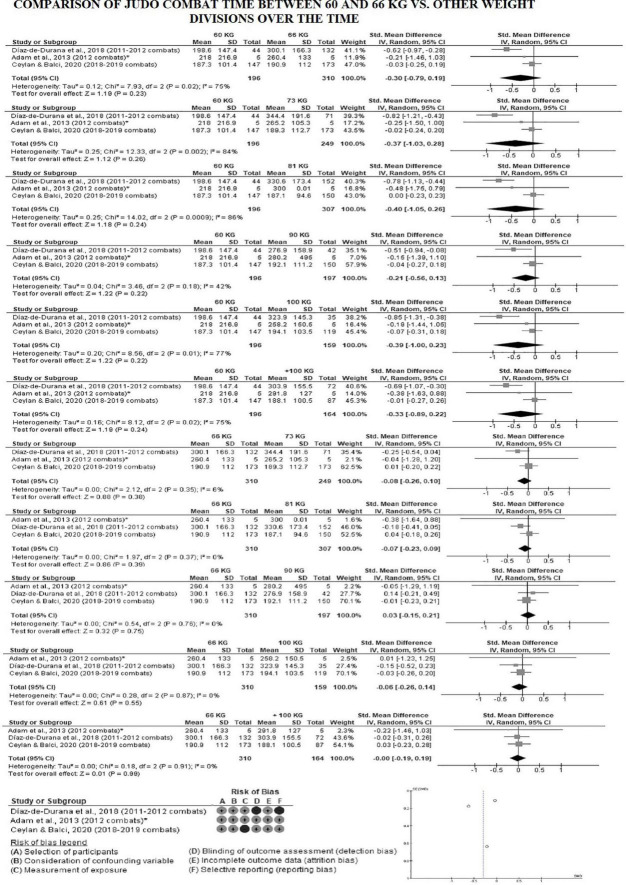
Combat time between lightweights vs. other weight division in male judo over the years. *Average combat time by category calculated by the authors of this study.

**FIGURE 6:**
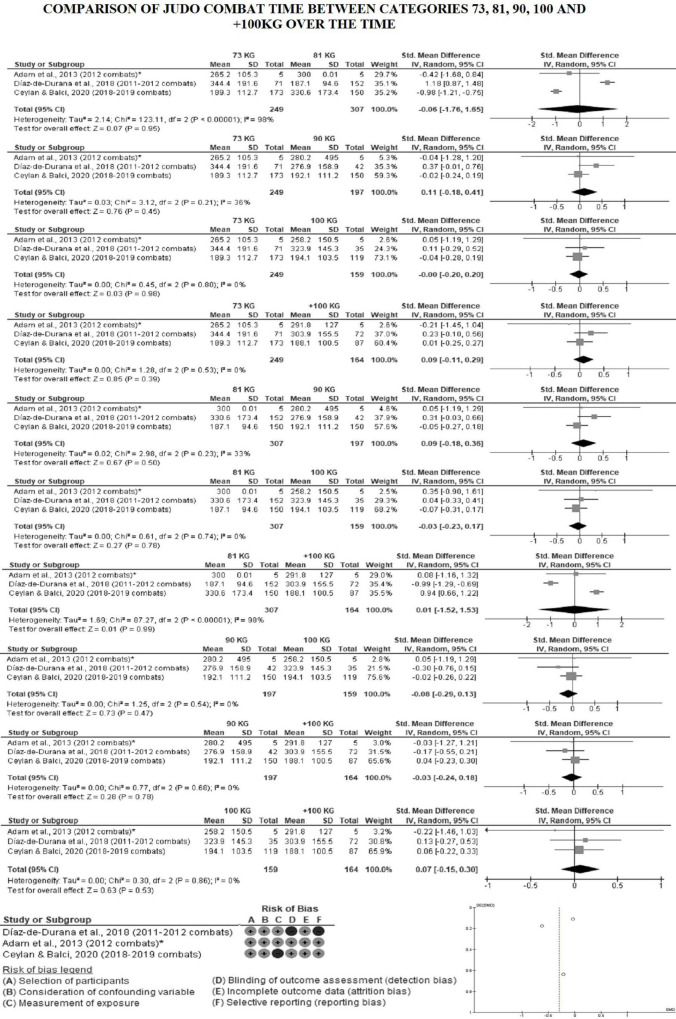
Combat time between middleweight and heavyweight in male judo over the years. *Average combat time by category calculated by the authors of this study.

Figure 4 shows the average combat time by weight division between 2010 and 2019. It was not possible to include some studies (Sterkowicz-Przybycien et al., 2017; Soriano et al., 2019) in the analysis by division, as data was presented as median and interquartile range (Sterkowicz-Przybycien et al., 2017) and they analyzed the divisions by grouping them (66 + 73 + 81 kg; 90 + 100 kg) (Sterkowicz-Przybycien et al., 2017; Soriano et al., 2019). In order to enter the data from Adam et al. (2013) in this analysis, we calculated the mean and standard deviation of the time in seconds of 5 combats from the champions in each weight division in the 2012 Olympics, which were reported in their study. The data indicates that combat time reduced and became more homogeneous by weight division over the years. Figures 5, 6 show the forest plot graphs of the combat time meta-analyses by division. No significant difference was found when comparing weight division between 2010 and 2019.

## Discussion

The purpose of this systematic review and meta-analysis was to synthesize literature data regarding the analysis of combat time of male judokas in international competitions between 2010 and 2019 and by weight division. Understanding how rule changes have affected the total time of male judo combats over years can help plan athletes’ training to achieve higher performance. The main results of this systematic review and meta-analysis showed that the average combat time of male judokas changed with each rule change (2010, 2013, 2017, and 2018), and the years 2011–2012 were those with the highest average combat time (Figure 3A). In addition, there was a tendency toward homogeneity in the combat time spent between the weight division over the years (Figure 4).

### Analysis of Total Male Judo Combat Time Between 2010 and 2019

Soriano et al. (2019) analyzed international combats from 2010 and found a total time of 202.8 s regarding the total combat time. Adam et al. (2013) and Barreto et al. (2019) analyzed combats from competitions between 2011 and 2012 and found 304.8 and 267.7 s, respectively (Figure 3A). Other reports with high-level athletes indicated a shorter female combat time in 2011–2012, with 232.7 ± 146.3 s (Miarka et al., 2016), and similar results in high-level international male athletes (Miarka et al., 2016a,b). These differences in the average male combat time between 2010 and 2011–2012 could be explained by the changes to the 2010 rules, which were in effect from January 2010 to December 2012. The main 2010 changes were reducing Golden Score time from 5 to 3 min, eliminating the *Koka* score, and prohibition of leg grip techniques (International Judo Federation, 2009). These changes had an impact on the athletes’ way of fighting. Existing time-motion and tactical analyses in judo research have provided preliminary data on the effects of these variables, such as modified visual search strategies to initiate gripping (Piras et al., 2014). Though, investigation of time-motion and tactical indicators without concern for weight category and competitive level would appear to deliver limited insight into the complex environment of judo (Adam et al., 2013). Indeed, the current findings corroborate preceding studies, suggesting that practical evaluation of high-level judo performance must account for the potential interaction of gripping strategies, subsequent attacks, and combat time (Calmet et al., 2010; Courel-Ibáñez et al., 2014; Miarka et al., 2018b; Kashiwagura et al., 2021).

Many athletes in 2010 might have been initially disqualified for performing techniques with leg grip techniques, which may have reduced combat time. Penalties could result from attempts to avoid the opponent’s control and feigning attack without intent to Score (false attack) (Escobar-Molina et al., 2014; Katicips et al., 2018; Martins et al., 2019). Additionally, when compared to previous tournaments, there was a significant decrease in scores by the penalty at the 2012 Olympics Games (0.44 penalties/min combat) when compared to the 2008 Olympic Games, with 1.26 penalties/min, and other international events (ranging from 1.46 to 2.17 penalties/min) (Heinisch et al., 2013; Miarka et al., 2016). Still, this was a tactical way to avoid a potential attack, and comparisons of the 2012 and 2013 European Championships presented an increase in the frequency of penalties, which was not reflected in scores (Franchini et al., 2013b). Over time, new strategies were learned, such as “scoring and maintain.” In this strategy the athlete stops attacking (and taking risks) after getting the first score, and managed the combat time leading the opponent to commit penalties, since at that time the punishments were equivalent to scores for the opponent. Thus, the athletes spent more time to finish the combat, however, they were taking less risk of receiving a projection and losing by *ippon*. The average combat time found by Adam et al. (2013) and Barreto et al. (2019) is close to the regular combat time of 5 min, which confirms the wide use of the mentioned strategy (Figure 3A). This form of fighting, in which the opponent’s punishment was prioritized rather than attacking and obtaining scores, was considered negative judo and it encouraged the adoption of new rules in 2013.

Therefore, the Golden Score time became unlimited in the 2013 and the punishments were no longer worth points; however, if the regular time ended with the combat in a draw, the athlete with the most punishment would lose. These rules were valid until 2016 (International Judo Federation, 2013). As we can see in Figure 3A, there was a reduction in combat time in 2016 to values close to 2010, with 237.4 s (Boguszewski, 2016). Thus, the 2013 rule changes should encourage athletes to attack more since penalties would only be helpful at the end of regular time (to tiebreaker) or the Golden Score. However, combat data from 2014 to 2015 showed that non-combativity was the most frequent violation of the rule (45.1%) and the one that most resulted in combat disqualification (49.3%), and leaving the combat area was in second place (13.4%) in the reasons for disqualification (Adam et al., 2018). Perhaps this reduction in combat time would be explained by greater technical efficiency, resulting in at the end of combat before the regular time. Blach et al. (2021) compared the attack efficiency index between the 2012 Olympic Games and the 2013 World Cup, and they observed higher attack efficiency values in 2013, although the statistical analysis did not show a significant difference (2012 = 5.92 vs. 2013 = 7.79, *p* = 0.083). In addition, as inducing the opponent to commit infractions was no longer a good strategy, as punishments no longer turned into scores (International Judo Federation, 2013), performing consecutive attacks with different grips became essential to surprise and defeat the opponent. Ito et al. (2019) observed that, in 2016 combats, re-gripping techniques resulted in more significant scoring rates than non-re-gripping techniques when competitors grip the dorsal region of the judogi or places other than the traditional grip (collar and sleeve). This makes sense, as Segedi et al. (2014) when analyzing the 2013 combats, found that 63.7% of them ended with Ippon. Furthermore, 97% of combats ended until regular, and only 3% needed a Golden Score to define the winner (Figure 3B).

There was another impactful rule change in 2017: the regular combat time for men was reduced from 5 to 4 min; the *Yuko* score was eliminated; the *Wazari* score became cumulative (2 *Wazari* were no longer equivalent to one *Ippon*); and punishments no longer decided the combat winner (except for the Golden Score) (International Judo Federation, 2017a). Then, a new rule change in 2018 determined that a punishment could also not decide the combat in the Golden Score, and the accumulation of 2 *Wazari* returned to equal the International Judo Federation, 2017b). Figure 3A shows that the duration of combats in the years 2018 and 2019 is shorter than in previous years, with 189.8 s (Ceylan and Balci, 2020). However, the number of combats that needed a Golden Score to define the winner increased after the 2017 rule changes (Figure 3B). While in 2013, only 3% of the combats needed the Golden Score to define the winner (Segedi et al., 2014), the 2018–2019 competitions had 21% of the combats requiring the Golden Score (Ceylan and Balci, 2020). There was a significant difference in this 17.7% increase in the occurrence of Golden Score (*p* = 0.03) in the years 2018–2019 compared to 2013 when we performed the meta-analysis (Figure 3B).

These data lead us to believe that male judokas are going through adapting to the new regular combat time of 4 min, which requires applying attacks with higher efficiency. As can be seen in Table 3, there was no major change in the total number of combats that ended before regular time (2013 = 63.2% vs. 2018–2019 = 64.7%), nevertheless the combats which ended in regular time had a huge difference in percentage between years (2013 = 33.6% vs. 2018–2019 = 14.4%). As in 2017, the regular time was reduced from 300 to 240 s, and athletes who were not yet used to the rule change needed the Golden Score time to define the winner. This means that most athletes might not have changed their combat strategies despite the reduced total combat time. Therefore, coaches should adapt their training to the new temporal demands of international male judo combat.

### Analysis of Total Male Combat Time by Weight Division Between 2010 and 2019

In addition to analyzing the total average time of male judo combats over the years, it is also important to identify whether there is a difference between the weight division. Therefore, training methods can be tailored to the specific needs of each group.

Data from Díaz-de-Durana et al. (2018) indicate that there were significant differences in total combat time between the weight divisions in 2011–2012 (*F* = 4.39; *p* ≤ 0.001; η^2^ = 0.049). The 60 kg category had a shorter combat time when compared to the other weight divisions (*p* ≤ 0.019), except for the middleweight (90 kg) (Figure 4). In other words, 60 kg athletes (who usually have dynamic, offensive, and effective combat) won the combat before the end of the regular time, which was 300 s (60 kg = 198.6 s) (Table 2). This characteristic of 60 kg athletes can be observed in the study of Adam et al. (2013), in which the 2012 Olympic Games 60 kg champion won all the combats by technical score (*Nage-waza* or *Ne-Waza*), there was no occurrence of victory by punishment, unlike what happened in other weight divisions. Furthermore, when analyzing the relative combat time (time of each movement/pause cycle), Díaz-de-Durana et al. (2018) found that the + 100 kg category had a longer relative combat time when compared to the other divisions (*p* ≤ 0.04; + 100 kg = 48.8 ± 28.1 vs. <60 kg = 28.7 ± 15.4; <66 kg = 35.8 ± 19.9; <73 kg = 35.5 ± 18.7; <81 kg = 34.4 ± 16.3; <90 kg = 37.2 ± 23.6 s), except for the half-heavyweight (<100 kg = 38.4 ± 32.9 s). Therefore, the + 100 kg category had fewer breaks during combat and spent more time in the movement cycle, which presupposes a more defensive and less dynamic combat. On the other hand, Sterkowicz-Przybycien et al. (2017), who also analyzed combats from 2011 to 2012, found no significant differences in combat time between weight divisions. However, they found a significant difference between categories when analyzing the relative combat time, similar to Díaz-de-Durana et al. (2018). The + 100 kg category spent more time compared to 60 kg category (+ 100 kg = 28.5 vs. 60 kg = 22.9 s; LL = –369.6; UL = –3.03) and to the combination of the intermediate weight division (+ 100 kg = 28.5 vs. 66 + 73 + 81 kg = 24.8 s; LL = –271.1; UL = –14.2) (Table 2).

In addition, a previous study (Ryszard et al., 2014) that analyzed athletes from the + 100 kg category during the 2012 Olympics observed that the attack efficiency indicator was higher in the 3rd and 4th min, with the 5th min and the Golden Score also showing greater attack efficiency than the first few minutes of combat (1st = 2.26; 2nd = 4.55; 3rd = 8.08; 4th = 8.47; 5th = 5.45; Golden Score = 5.26). Furthermore, as combat time progressed, the number of backward attacks (throws by leg) was reduced, and forwarding attacks increased. Preceding reports, which indicated that heavyweight athletes may adopt the previously reported strategy of extending the time spent gripping in order to maintain dominant positioning and delay attacks until later in the match or prompt penalties to be committed by their opponents (Courel-Ibáñez et al., 2014; Escobar-Molina et al., 2014; Sterkowicz-Przybycien et al., 2017), whereas lighter athletes may need to more frequently alter their gripping patterns in order to engage their opponents (Miarka et al., 2012, 2016b; Courel-Ibáñez et al., 2014).

Regarding weight division differences, preceding studies indicated that varying throwing techniques while maintaining consistent grip positions is likely beneficial for all athletes regardless of weight category (Courel-Ibáñez et al., 2014). Past research indicated biomechanical differences in female and male combats (Soto et al., 2020a). The male middleweight category had more effective attacks with arm and leg levers than lighter and heavier judokas. Lower extremity techniques (i.e., ashi-waza) executed to the front or side (left or right), such as o-soto-gari and the ashi-harai, require high amounts of relative torque/weight before realizing the opponent’s imbalance (kuzushi), compared to those executed to the rear orientation which involves rotation such as morote-seoi-nage, and sutemi-waza, such as tomoe-nage (Soto et al., 2020). An advantage that physiological and biomechanical variables have is that they can be measured directly rather than indirectly, as is often the case with isolated psychological variables (Tod et al., 2015). The challenge may be the selection of appropriate measures. For example, judo is a multidimensional construct consisting of numerous psychological and physiological components, some of which may be relevant and others irrelevant to the physical performance (Tod et al., 2015). While potential data limitations exist due to studies’ internal validation requirements, the procedures utilized reflect a practical application to observe critical events in combat time, which can be quantified consistently and reliably (e.g., Sterkowicz-Przybycien et al., 2017). We highlight that the present study’s main limitation is that the meta-analyses have few studies. These meta-analyses showed the effect size between categories and studies did not provide experimental data or control situations. The present research suggests more studies, considering experimental data and control situations, using combat phases, technical-tactical variables, and decision-making.

As previously mentioned, there was an impactful change in the rules in 2017 and the regular combat time was reduced to 240 s (International Judo Federation, 2017b). This rule change reduced the average combat time in 2018–2019 as expected (Figure 3A); however, there was homogeneity in the total combat time between the categories in 2018–2019, which did not occur before (Figure 4). In fact, Ceylan and Balci (2020) found no significant difference between divisions [*F*(6, 1657) = 0.36; *p* = 0.90; η^2^ = 0.001]. This indicates that the 2017 rule changes caused variation in behavior between weight division compared to previous years, which caused homogeneity in the use of combat time. These behavior changes must have occurred in the last minute of the regular combat time and/or in the Golden Score, since there was no higher variation in the percentage of combats that ended before regular time comparing 2013 (63.2%) vs. 2018–2019 (64.7%). Confirming this line of thought, data from Segedi et al. (2014) show that the 73 kg category had the highest occurrence of Golden Score in 2013 (11.8%) (Table 3), while in 2018–2019, Ceylan and Balci (2020) reported the highest occurrence of Golden Score was in the 66 kg category (they did not inform the value). However, Ceylan and Balci (2020) still found a significant difference in the relationship between weight division and end-of-combat time in 2018–2019 (*p* < 0.001). Furthermore, no significant difference was found in computing a meta-analysis of total male combat time data between divisions over years from the studies by Adam et al. (2013), Díaz-de-Durana et al. (2018), and Ceylan and Balci (2020) (Figures 5, 6). More studies need to be carried out to better verify what is happening in each combat phase between the different weight divisions after the 2017 rule change. The temporal characteristics reported in this meta-analysis throughout a judo match provide information that can be used in both program design and the manipulation of training variables of high-level athletes and weight categories (Miarka et al., 2020a). Although examination of the mechanisms underlying the cognitive strategy and judo performance relationship may yield valuable data, such research might help coaches, athletes, trainers, and sports medicine staff to cognitive strategies associated with specific ends (Miarka et al., 2018b), studies need to employ data collection and analysis designs allowing adequate analysis (Tod et al., 2015).

## Conclusion

This systematic review and meta-analysis aimed to synthesize literature on male judo combat time in international competitions between 2010 and 2019 and by weight division. We observed significant changes in the male judo combat time with each rule change (2010, 2013, 2017, and 2018). As one implication, cognitive strategies may contribute to the reliability of judo rules associated with their respective goals. Regarding the weight divisions, there were differences between the 60 and + 100 kg categories and the other categories in the years 2011–2012. At that time, lighter athletes spent less combat time than other categories, and heavier judokas spent more relative combat time than other categories. In other words, the extreme categories had different time demands to define the combat champion, and they likely had different behaviors to achieve that goal. However, no significant difference was found between the combat time of the weight divisions after the 2017 rule changes, although there were still differences concerning the end of the combats. If combat time influences tactical performance in each weight category, then providing athletes with a prescribed cognitive and behavior strategy to follow may help to standardize psychological factors that might otherwise contribute to judo.

These results indicate that new studies need to be carried out to identify new temporal behaviors in judo with each new change in the rules, which usually occurs at each Olympic cycle. We realized that the data from the included studies point to changes toward homogeneity of the average combat time spent between weight divisions over the years. However, there was also an increase in the occurrence of the Golden Score, which can determine differences between groups of athletes in the same category: those who finish the combat until regular time and those who need a Golden Score to define the winner. From the knowledge of how athletes behave in a new set of rules, coaches can plan training to meet the specific energy demands of athletes by weight division and create combat strategies based on time-motion combat phases. More studies need to be conducted analyzing male combats separated by weight division.

## Data Availability Statement

The original contributions presented in the study are included in the article/supplementary material, further inquiries can be directed to the corresponding author/s.

## Author Contributions

LB participated in the study design, search and selection of articles, data collection and analysis, and manuscript preparation. MAS and LF participated in the search and selection of articles and data collection and analysis. DV and FM participated in the data analysis and manuscript preparation. BM and CB participated in the study design, data analysis, and manuscript preparation. All authors contributed to the article and approved the submitted version.

## Conflict of Interest

The authors declare that the research was conducted in the absence of any commercial or financial relationships that could be construed as a potential conflict of interest.

## Publisher’s Note

All claims expressed in this article are solely those of the authors and do not necessarily represent those of their affiliated organizations, or those of the publisher, the editors and the reviewers. Any product that may be evaluated in this article, or claim that may be made by its manufacturer, is not guaranteed or endorsed by the publisher.
